# Effects of an opioid-free care pathway vs. opioid-based standard care on postoperative pain and postoperative quality of recovery after laparoscopic bariatric surgery: A multicentre randomised controlled trial

**DOI:** 10.1097/EJA.0000000000002193

**Published:** 2025-05-14

**Authors:** Alexander Olausson, Pether Jildenstål, Paulin Andréll, Eva Angelini, Erik Stenberg, Ville Wallenius, Henrik Öhrström, Sven-Egron Thörn, Axel Wolf

**Affiliations:** From the Institute of Health and Care Sciences, Sahlgrenska Academy, University of Gothenburg (AO, PJ, EA, AW), Department of Health Sciences, Lund University (PJ), Department of Anesthesiology and Intensive Care, Skåne University Hospital, Lund (PJ), Department of Anaesthesiology and Intensive Care Medicine, Institute of Clinical Sciences, Sahlgrenska Academy, University of Gothenburg (PA, S-ET), Department of Anaesthesiology and Intensive Care Medicine/Pain Centre, Sahlgrenska University Hospital, Region Västra Götaland, Gothenburg (PA), Department of Surgery, Faculty of Medicine and Health, Örebro University, Örebro (ES), Department of Surgery, Institute of Clinical Sciences, Sahlgrenska Academy, University of Gothenburg (VW), Department of Surgery, Sahlgrenska University Hospital/Östra, Region Västra Götaland, Gothenburg (VW), Department of Anesthesiology and Intensive Care, Örebro University Hospital, Region Örebro län, Örebro (HÖ), Department of Anesthesiology and Intensive Care, Lindesberg Hospital, Region Örebro län, Lindesberg (HÖ), Department of Anaesthesiology and Intensive Care Medicine, Sahlgrenska University Hospital/Östra, Region Västra Götaland, Gothenburg, Sweden (S-ET, AW), Institute of Nursing and Health Promotion, Oslo Metropolitan University, Oslo, Norway (AW) and Centre for Person-Centred Care (GPCC), University of Gothenburg, Sweden (AW)

## Abstract

**BACKGROUND:**

Opioid-free anaesthesia (OFA) may enhance postoperative recovery after bariatric surgery, but its combined effect with opioid-free interventions has not been studied.

**OBJECTIVE(S):**

To compare postoperative pain and recovery after laparoscopic bariatric surgery with a total opioid-free care pathway and conventional opioid-based treatment.

**DESIGN:**

A multicentre nonblinded controlled trial.

**SETTING:**

Two university hospitals in Sweden.

**PATIENTS:**

Adult patients scheduled for laparoscopic bariatric surgery were enrolled between May 2019 and November 2023. Of 837 patients screened, 112 were randomised, and 110 were included in the analysis: 55 in the intervention and 55 in the control group.

**INTERVENTION(S):**

Patients were randomised to an opioid-based standard care (control group) or to an opioid-free care pathway (intervention group), including intraoperative OFA and postoperative first-line transcutaneous electrical nerve stimulation (TENS) treatment.

**MAIN OUTCOME MEASURES:**

The primary outcome was the change in patient-reported postoperative pain intensity on a numerical rating scale (NRS) from arrival in the postanaesthesia care unit (PACU) until discharge to the surgical ward. Key secondary outcomes were postoperative pain intensity, in-hospital opioid consumption, and postoperative quality of recovery scale (PQRS) scores.

**RESULTS:**

There was no difference between the groups regarding the changes in pain intensity from arrival in PACU until discharge to the ward, with mean ± SD changes in NRS of 3.20 ± 3.01 (intervention) vs. 3.15 ± 2.25 (control); mean difference (MD) 0.04 [(95% confidence interval (CI), −1.00 to 1.08); *P* = 0.97], and pain intensity at 24 h (*P* = 0.078), 72 h (*P* = 0.060), and 3 months (*P* = 0.30) postoperatively. The intervention group had a significantly lower opioid consumption in the PACU; mean morphine equivalents 6.08 ± 12.31 vs. 51.1 ± 14.9 mg; MD −45.0 (95% CI, −50.1 to −39.8) mg; *P* < 0.0001; and during the hospital stay MD −40.3 (95% CI, −54.4 to −25.9) mg; *P* < 0.0001. Total PQRS scores did not differ significantly over the 3-month follow-up.

**CONCLUSION:**

The opioid-free care pathway offers patients pain relief and recovery outcomes comparable to conventional opioid-based care and reduces opioid use after laparoscopic bariatric surgery.

**TRIAL REGISTRATION:**

ClinicalTrials.gov NCT03756961.

**KEY POINTS**

- The effects of an opioid-free care pathway that combines both pharmacological and nonpharmacological opioid-free interventions have not yet been studied.
- An opioid-free care pathway can provide comparable pain relief and quality of recovery outcomes with opioid-based standard treatment for up to 3 months after surgery, while significantly reducing the in-hospital opioid consumption.
- The novel approach of combining intraoperative opioid-free anaesthesia with postoperative transcutaneous electrical nerve stimulation (TENS) as first-line pain treatment has the potential to eliminate the use of perioperative opioids for patients undergoing bariatric surgery.

## Introduction

Bariatric surgery is a highly effective treatment for people with severe obesity, leading to substantial and sustainable weight loss, reducing obesity-related comorbidities and increasing health-related quality of life.^1–3^ However, due to the reliance on opioids as the standard treatment for general anaesthesia and postoperative pain management, patients may face substantial perioperative challenges when undergoing bariatric surgery and are at higher risk of respiratory depression,^4^ postoperative nausea and vomiting (PONV),^5^ insufficient pain control and problematic opioid use.^6–8^ Opioid-related side effects worsen early postoperative recovery and can impair long-term recovery and patient outcomes if not adequately addressed.

The Enhanced Recovery After Bariatric Surgery guidelines^9^ recommend opioid-sparing and nonopioid pain management strategies to enhance postoperative recovery. Opioid-free anaesthesia (OFA) reduces postoperative side effects without having a negative impact on patient safety.^10–12^ In bariatric surgery, OFA may also improve pain outcomes in the early postoperative phase^11^ and yield similar perioperative experiences to opioid-based anaesthesia (OBA).^13^ Nevertheless, data on OFA are inconclusive and long-term patient recovery has been poorly explored, thus, opioids remain the standard of perioperative care.

To date, no study has implemented an opioid-free intervention that encompasses both the intraoperative and postoperative phases. Typically, there is a transition to standard postoperative treatment protocols although opioid-sparing strategies are warranted following bariatric surgery.^9^ Transcutaneous electric nerve stimulation (TENS) combines pain-relieving properties with an opioid-sparing effect for acute postoperative pain in various contexts, including laparoscopic surgery.^14,15^ Combining OFA with postoperative TENS treatment could enhance postoperative patient outcomes and further reduce the need for opioids perioperatively. This randomised controlled trial (RCT) aimed to compare the effects of a total opioid-free care pathway and conventional opioid-based treatment on postoperative pain and recovery after laparoscopic bariatric surgery.

## Methods

This study captures data from the first phase of a multicentre, nonblinded, RCT (ClinicalTrials.gov NCT03756961) conducted at two university hospitals in Sweden between May 2019 and November 2023.

### Ethics

The clinical trial was approved by the Swedish Ethical Review Board (DNR 1006-17; Chairperson Birgitta Henriksson) on 26 February 2018 and the Swedish Medical Products Agency (EU CT 2023-505934-86-00). All patients received written and oral information. Written informed consent was obtained from all participants. The clinical trial was overseen by an independent data and safety monitoring board at both study sites, which reviewed safety data and ensured the trial's conduct adhered to the Declaration of Helsinki and Good Clinical Practice guidelines. The study follows the Consolidated Standards of Reporting Trials (CONSORT) guidelines.

### Patients

The inclusion criteria were patients aged at least 18 years, scheduled for laparoscopic gastric bypass or sleeve gastrectomy at one of the two study sites, and informed consent. The exclusion criteria are outlined in Table 1. Patients were screened for eligibility via medical records and asked to participate during the preoperative assessment visit at the hospital. Screening, recruitment and enrolment were conducted by the study nurse and principal investigator at each site.

**Table 1:** Exclusion criteria

1. American Society of Anesthesiologists (ASA) classification greater than III
2. Cardiovascular disease with bradycardia (heart rate <50 bpm)
3. Serious liver disease
4. Insufficient knowledge of the Swedish language
5. Untreated serious psychiatric disease
6. Neurocognitive dysfunction
7. Pregnancy or women of childbearing age who are not using contraception and have not taken a pregnancy test before surgery
8. Malignant disease with an expected short survival
9. Chronic pain treated with opioids
10. Substance abuse
11. Hypersensitivity to oxycodone, esketamine, dexmedetomidine or lidocaine
12. Presence of a pacemaker or implantable cardioverter–defibrillator (ICD)
13. Inability to complete questionnaires
14. Refusal to participate in the study

### Randomisation and interventions

Patients were randomised to the intervention or the control group in a 1 : 1 ratio on the day of surgery by the study nurse using computerised allocation (Microsoft Access program). The randomisation was stratified by study site and balanced by sex, age and body mass index as minimisation variables. The group allocation was revealed to the patient and the clinical staff involved. The study personnel collected data throughout the surgery, in the postanaesthesia care unit (PACU), and during the 3-month follow-up.

### Primary outcome

Pain intensity was measured by the study nurse at the bedside in PACU using a numerical rating scale (NRS 0 to 10, 0 = no pain up to 10 = worst imaginable pain). The primary outcome was a comparison between the two groups in the change of NRS between arrival in the PACU and at the time of discharge to the surgical ward.

### Secondary outcomes

The secondary outcomes were differences in patient-reported pain intensity (NRS) after PACU discharge, in-hospital opioid consumption and total recovery after surgery measured using the Postoperative Quality of Recovery Scale (PQRS). Additional outcomes included the need for opioid analgesia, time spent in the PACU and the number of participants reporting a pain intensity rated NRS less than 3 at rest.

Pain intensity (NRS) after PACU discharge was assessed in the surgical ward (24 h), the first day at home (72 ± 24 h) and 3 months postoperatively. Pain assessment was conducted by the study nurse at the bedside during the in-hospital stay and by telephone after hospital discharge. Opioid consumption was obtained from medical records and converted to oral morphine equivalents in milligrams.^7,16^

The PQRS was administered following the preoperative assessment and study inclusion (baseline), in the PACU (20 and 40 min), in the surgical ward (24 h), first day at home (72 ± 24 h) and 14, 30 and 90 days postoperatively. The PQRS includes physiological, nociceptive, emotional, cognitive, activities of daily living and overall patient perspective domains, along with subsequent parameters in each domain.^17^ The study used the Swedish translation of the PQRS.^18^ PQRS scores were calculated as described in Supplement 1. Data collection was performed bedside during the in-hospital stay, and remotely after discharge. An electronic patient-reported outcome (ePRO) system (TrialOnline, Replior AB) was used beyond the 14-day follow-up for all parameters except the cognitive domain, which was assessed through a telephone call.

Safety data including adverse events, serious adverse events and suspected unexpected serious adverse events were recorded from admission until discharge from the hospital. Complications were presented according to the Clavien–Dindo classification.^19^

### Anaesthetic technique

**Fig. 1:**
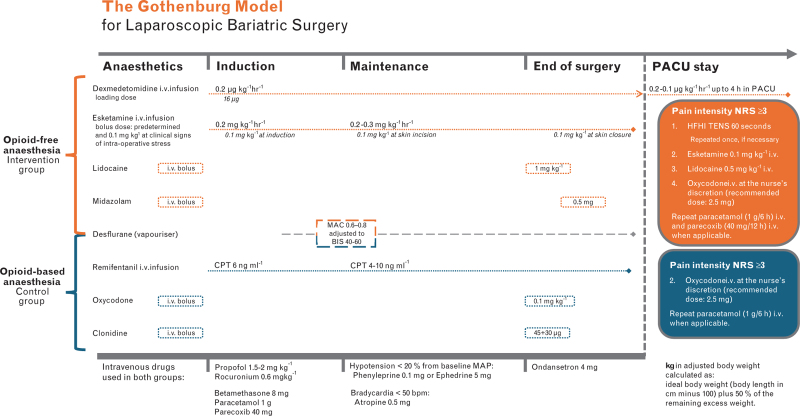
Pharmacological and non-pharmacological regimens for general anaesthesia and postoperative pain management.

In the operating theatre, standard monitoring was applied, and pre-oxygenation was performed following standard guidelines for all patients. An automatic lung recruitment manoeuvre was performed postintubation. Pharmacological regimens for general anaesthesia and postoperative pain management are presented in Fig. 1.

Pre-operatively, patients in the intervention group received brief instruction on TENS use. Upon arrival in the PACU, patients reporting pain intensity rated NRS at least 3 initially received high-frequency (80 Hz), high-intensity (40 mA) transcutaneous electrical nerve stimulation (HFHI TENS) administered by the nurse.^15,20^ The TENS electrodes were placed with a minimum distance of 3 cm and a maximum of 30 cm between them, ensuring effective stimulation across the dermatomes involved. HFHI TENS was applied for 1 min and repeated once if the analgesic effect was inadequate (i.e. NRS ≥ 3). If TENS treatment did not result in adequate pain relief, the pharmacological pain management algorithm was followed (Fig. 1). Once patients attained an acceptable level of consciousness in the PACU, they received instruction on the self-administered TENS programme, involving modulated pulse duration stimulation at an intensity chosen by the patient. In the surgical ward, nurse-administered and self-administered TENS treatment were continued. The control group received a multimodal, opioid-based standard care regimen for anaesthesia and postoperative pain management.

### Statistical analysis

This study was designed as a noninferiority trial for its primary outcome and a superiority trial for its secondary outcomes. The main analyses were performed on the modified intention-to-treat (mITT) population, comprising participants who initiated treatment after randomisation, with complementary analyses on the per-protocol population, including participants without protocol deviations. The primary outcome, defined as the pain intensity change from PACU arrival to discharge with a margin of −1 on the NRS score, was assessed based on a power calculation for noninferiority with a standard deviation of 1.62. To achieve 80% power at a 0.05 significance level, a sample size of 43 patients per group was needed. To account for potential dropouts, the trial enrolled 55 patients per group. Noninferiority was confirmed if the lower limit of the two-sided 95% confidence interval (CI) exceeded −1 for the unadjusted mean difference in the pain intensity score change between groups.

Continuous variables were analysed using Fisher's nonparametric permutation test, while ordered categorical variables and dichotomous variables were analysed with the Mantel–Haenszel *χ*^2^ test and Fisher's exact test, respectively. Changes in continuous variables over time between the two groups were examined with an analysis of covariance (ANCOVA) adjusted for baseline values. Within-group comparisons were conducted using Fisher's nonparametric permutation test for paired observations for continuous variables and the Sign test for dichotomous and ordered categorical variables. For time-to-event data, the log-rank test was used, and Kaplan–Meier graphs were created to illustrate the results.

The main results are presented as mean differences (MD) between groups, with 95% CI, *P* values and effect size. Descriptive statistics for continuous variables include mean ± SD, median (range), while categorical variables are presented as numbers (%). All statistical analyses were conducted using SAS (Version 9.2, SAS Institute Inc., Cary, North Carolina, USA). All statistical tests were two-sided and conducted at the 5% significance level.

## Results

### Patient characteristics

**Fig. 2:**
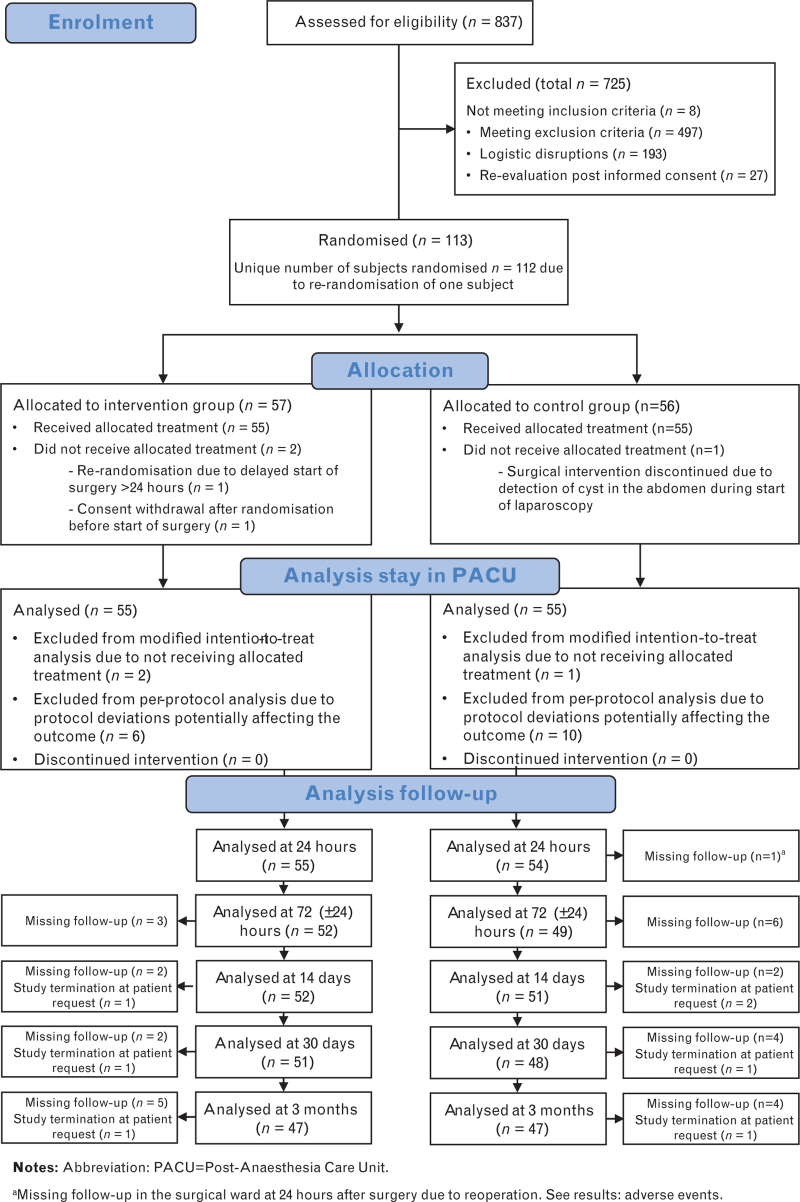
Study flow chart.

Of the 837 patients screened for eligibility between May 2019 and November 2023, 112 underwent randomisation, and 110 received the allocated treatment: 55 in the intervention group, 55 in the control group. Sixteen participants were excluded from the per-protocol analysis: 6 from the intervention and 10 from the control group. During the 3-months follow-up, three participants in the intervention and four in the control group terminated their study participation (Fig. 2). Baseline characteristics and perioperative medications are presented in Table 2; baseline characteristics were comparable between the groups.

**Table 2:** Patient baseline characteristics and perioperative medications

Variable	Intervention group (*n* = 55)	Control group (*n* = 55)
Demographics		
Female sex, (*n* (%))	41 (74.5)	40 (72.7)
Age (years)	39.1 ± 9.7 37 [22 to 57]	38.5 ± 11.2 36 [22 to 71]
BMI (kg m^−2^)	42.8 ± 4.9 42.3 [33 to 55.2]	42.8 ± 4.9 43.3 [30.5 to 58.9]
University hospital site 1	26 (47,3)	27 (49)
University hospital site 2	29 (52,7)	28 (51)
Laparoscopic gastric by-pass	38 (69.1)	36 (65.5)
Laparoscopic sleeve gastrectomy	17 (30.9)	19 (34.5)
Education		
Primary school (9 years), (*n* (%))	55 (100)	55 (100)
Upper secondary education, (*n* (%))	52 (94.5)	50 (90.9)
University degree, (*n* (%))	15 (27.3)	21 (38.2)
ASA physical status classification, (*n* (%))		
Class I	4 (7.3)	8 (14.5)
Class II	36 (65.5)	27 (49.1)
Class III	15 (27.3)	20 (36.4)
Comorbidity, (*n* (%))		
Diabetes, type I and II	10 (18.2)	2 (3.6)
Hypertension	6 (10.9)	5 (0)
Hypothyroidism	5 (9)	5 (9)
Obstructive sleep apnoea	5 (9)	3 (5.4)
Hyperlipidaemia	5 (9)	0 (0)
Asthma	3 (5.4)	2 (3.6)
Depressive disorders	3 (5.4)	7 (12.7)
Anxiety disorders	3 (5.4)	3 (5.4)
Intraoperative medications		
Dexmedetomidine infusion (μg)	53 [21.1 to 105.6]	0 [0 to 0]
Esketamine infusion (mg)	75.4 [44 to 140]	0 [0 to 0]
Esketamine (mg)	36.4 [6 to 90.8]	0 [0 to 0]
Lidocaine (mg)	94 [70 to 200]	0 [0 to 0]
Remifentanil total infusion (μg)	0 [0 to 0]	1312 [629 to 3566]
Postoperative medications in PACU		
Dexmedetomidine infusion (μg)	53 [21.1 to 105.6]	0 [0 to 0]
Esketamin (mg)	9.5 [0 to 80] *n* = 35	10.1 *n* = 1^a^
Lidocaine (mg)	80 [4.5 to 260] *n* = 31	0 [0 to 0]

Data are presented as mean ± SD, median [range], or *n*(%). The number in the bottom row of columns indicates deviations from the total group size. BMI, body mass index; PACU, post-anaesthesia care unit.

a Esketamine was administered to one participant in the control group at the discretion of the principal investigator due to insufficient analgesic effect of opioids.

### Primary outcome

There was no difference between the groups regarding the change in pain intensity from PACU arrival to discharge with mean ± SD changes in NRS of 3.20 ± 3.01(intervention) vs. 3.15 ± 2.25 (control); mean difference (MD) 0.04; 95% confidence interval (CI), −1.00 to 1.08, *P* = 0.97. The result remained nonsignificant when adjusted for baseline pain intensity and minimisation variables: MD 0.26 (95% CI −0.35 to 0.88), and in the per-protocol analysis: MD −0.33 (95% CI −1.43 to 0.77) (Table 3).

**Table 3:** Postoperative pain, opioid consumption and length of stay in the PACU data

Variable	Intervention group (*n* = 55)	Control group (*n* = 55)	Mean difference (95% CI)	*P* value	Effect size
Change in pain intensity from arrival to discharge from the PACU, NRS	3.20 ± 3.01 3 [−4 to 10] *n* = 51	3.15 ± 2.25 3 [−2 to 9] *n* = 52	0.04 (95% CI −1.00 to 1.08)	0.97	0.02
Change in pain intensity from arrival to discharge from the PACU, NRS^a^	Adj 3.31 ± 3.0	Adj 3.04 ± 2.25	0.26 (95% CI −0.35 to 0.88)	Adj 0.40	Adj 0.02
Change in pain intensity from arrival to discharge from the PACU, NRS^b^	3.02 ± 2.75 3 [−4 to 8] *n* = 45	3.36 ± 2.39 3 [−2 to 9] *n* = 42	−0.34 (95% CI −1.44 to 0.77)	0.57	0.13
Change in pain intensity from arrival to discharge from the PACU, NRS^c^	Adj 3.20 ± 2.75	Adj 3.16 ± 2.39	0.04 (95% CI −0.59 to 0.68)	Adj 0.89	Adj 0.13
Pain intensity at baseline before surgery, NRS	0.39 ± 1.25 0 [0 to 6] *n* = 54	0.84 ± 1.65 0 [0 to 7] *n* = 55	−0.45 (95% CI −1.00 to 0.10)	0.13	0.30
Maximum pain intensity within the first 30 min after arrival to the PACU, NRS	6.26 ± 2.98 7 [0 to 10] *n* = 53	6.55 ± 1.83 6 [1 to 10] *n* = 55	−0.28 (95% CI −1.21 to 0.67)	0.58	0.11
Pain intensity at discharge from the PACU, NRS	3.06 ± 1.89 3 [0 to 8] *n* = 52	3.33 ± 1.63 3.5 [0 to 8] *n* = 52	−0.27 (95% CI −0.96 to 0.42)	0.47	0.15
Pain intensity < 3 in NRS before discharge from the PACU, *n* (%)	15 (27.3%)	6 (10.9%)	16.4 (95% CI 0.2 to 32.5)	0.051	0.43
Maximum pain intensity during the first 24 h in hospital, NRS	6.80 ± 2.55 8 [0 to 10] *n* = 55	6.76 ± 1.63 7 [1 to 10] *n* = 55	0.04 (95% CI −0.77 to 0.85)	0.96	0.02
Pain intensity after 24 h, NRS	2.48 ± 1.78 2.5 [0 to 8] *n* = 54	3.15 ± 2.00 3 [0 to 8] *n* = 54	−0.67 (95% CI −1.40 to 0.05)	0.078	0.35
Pain intensity after 72 (±24) hours, NRS	1.60 ± 1.45 1 [0 to 6] *n* = 48	2.24 ± 1.67 2 [0 to 7] *n* = 45	−0.64 (95% CI −1.29 to −0.00)	0.060	0.41
Pain intensity after 3 months, NRS	0.16 ± 0.74 0 [0 to 4] *n* = 45	0.40 ± 1.18 0 [0 to 6] *n* = 45	−0.24 (95% CI −0.65 to 0.15)	0.30	0.25
Opioid consumption during the PACU stay, ME in milligram	6.08 ± 12.31 0 [0 to 75] *n* = 55	19.1 ± 13.6 15 [0 to 63] *n* = 55	−13.1 (95% CI −17.9 to −8.1)	<0.0001	1.01
Opioid consumption during the PACU stay including preemptive dose of oxycodone, ME in milligram	6.08 ± 12.31 0 [0 to 75] *n* = 55	51.1 ± 14.9 52.5 [27 to 87] *n* = 55	−45.0 (95% CI −50.1 to −39.8)	<0.0001	3.29
Opioid consumption during entire hospital stay, ME in milligram	18.2 ± 44.6 0 [0 to 292.5] *n* = 55	58.4 ± 32.2 55.5 [0 to 126] *n* = 55	−40.3 (95% CI −54.4 to −25.9)	<0.0001	1.03
Length of stay in the PACU, minutes	137.7 ± 56.4 120 [60 to 333]	131.4 ± 74.0 105 [60 to 452]	6.24 (95% CI −18.52 to 31.12)	0.63	0.09

Data are presented as mean ± standard deviation and median [range], *n* = number, and mean difference (MD) with 95% confidence interval (CI). The number in the bottom row of columns indicates deviations from the total group size. For the comparison between groups, Fisher's nonparametric permutation test was used for continuous variables. The confidence interval for the mean difference between groups is based on Fisher's nonparametric permutation test. Adj, adjusted; ME, morphine equivalents; NRS, numerical rating scale (0 to 10); PACU, post-anaesthesia care unit.

a Sensitivity analysis adjusted for pain intensity at baseline before surgery and the minimisation variables study site, sex, age and body mass index (BMI) using Analysis of Covariance (ANCOVA).

b Per protocol analysis.

c Sensitivity analysis on the per protocol population adjusted for baseline pain and the minimization variables study site, sex, age and body mass index (BMI) using ANCOVA.

### Secondary outcomes

There was no difference in postoperative pain intensity (NRS) between the intervention and the control group at 24 h (*P* = 0.078), 72 (±24) h (*P* = 0.060) and 3 months (*P* = 0.30) postoperatively (Table 3). Twenty patients (36%) in the intervention group and 50 patients (90%) in the control group required opioids during the PACU stay; the mean consumption of opioids (morphine equivalents) was significantly lower in the intervention group vs. the control group: 6.08 ± 12.31 vs. 51.1 ± 14.9 mg; MD −45.0 (95% CI, −50.1 to −39.8) mg; *P* < 0.0001 (Table 3). The result was consistent after excluding the control group's pre-emptive postoperative dose of intravenous oxycodone: 6.08 ± 12.31 vs. 19.1 ± 13.6 mg; MD −13.1 (95% CI, −17.9 to −8.1) mg; *P* < 0.0001. The total opioid consumption during the in-hospital stay remained significantly lower in the intervention group vs. the control group: MD −40.3 (95% CI, −54.4 to −25.9) mg; *P* < 0.0001. There was no difference in time spent in the PACU (*P* = 0.63). By discharge from the PACU, 15 (27%) participants in the intervention group and six (10.9%) in the control group reported pain intensity rated NRS less than 3 (*P* = 0.051) (Table 3).

**Fig. 3:**
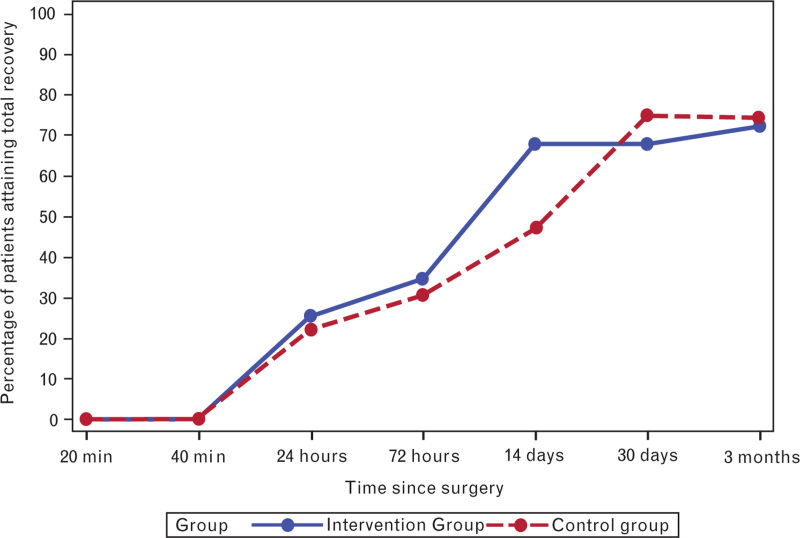
Total postoperative quality of recovery in all Postoperative Quality of Recovery Scale domains.

**Fig. 4:**
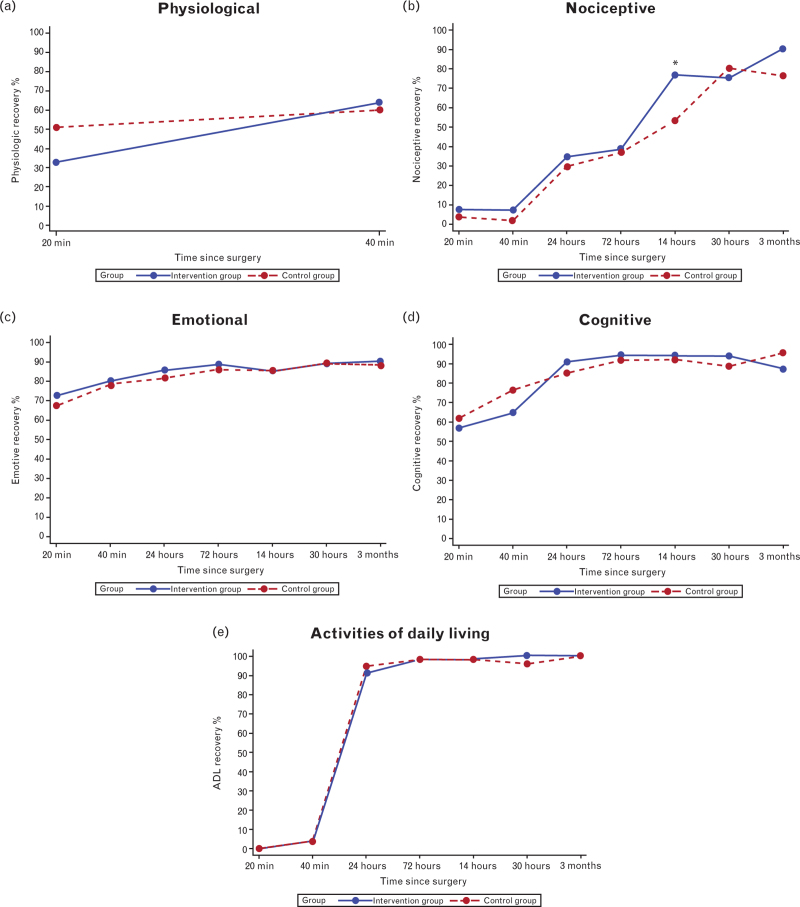
PQRS domains: physiological, nociceptive, emotional, cognitive and activities in daily living (ADL).

**Fig. 5:**
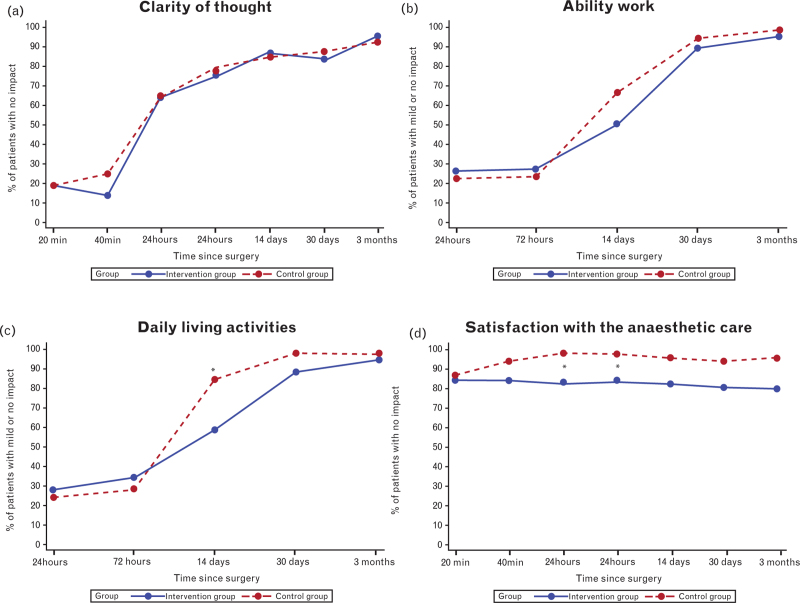
Overall patient perspective in the Postoperative Quality of Recovery Scale (PQRS).

PQRS scores improved over time in both groups, with no statistically significant differences between groups at any postoperative assessment (Table 4 and Figs. 3–5). At the 3-month follow-up, 72.3% in the intervention group and 74.5% in the control group achieved total recovery (Table 4 and Fig. 3). A significant difference was noted in the PQRS nociceptive domain at 14 days postoperatively, with 23.4% more participants in the intervention group achieving nociceptive recovery (*P* = 0.030) (Fig. 4). However, a breakdown in the nociceptive domain showed no significant difference between groups for the included parameters postoperative pain (*P* = 0.17) and PONV (*P* = 0.61) at 14 days.

**Table 4:** Total postoperative quality of recovery during stay in post-anaesthesia care unit and at follow-up

Postoperative measurement time points, *n* (%) recovered	Intervention group (*n* = 55)	Control group (*n* = 55)	Mean difference ()	*P* value
20 min	0 (0) *n* = 55	0 (0) *n* = 55		
40 min	0 (0) *n* = 55	0 (0) *n* = 55		
24 h	14 (25.5) *n* = 55	12 (22.2) *n* = 54	3.2 (−14.6 to 21.1)	0.86
72 (+/- 24) hours	18 (34.6) *n* = 52	15 (30.6) *n* = 49	4.0 (−16.2 to 24.3)	0.83
14 days	36 (67.9) *n* = 53	24 (47.1) *n* = 51	20.9 (0.4 to 41.4)	0.051
1 month	34 (68.0) *n* = 50	36 (75.0) *n* = 48	−7.0 (−26.9 to 12.9)	0.59
3 months	34 (72.3) *n* = 47	35 (74.5) *n* = 47	−2.1 (−22.1 to 17.9)	1.00

Number and percentage (%) of patients returning to baseline values or improved across all dimensions in the Postoperative Quality of Recovery Scale (PQRS) at each measurement time point after surgery. For each time point, the bottom-line number indicates the number of patients with available data, accounting for missing data in long-term follow-up. For the comparison between groups, Fisher's exact test (lowest 1-sided *P v*alue multiplied by 2) was used. The confidence interval for dichotomous variables is the unconditional exact confidence limits. PACU, post-anaesthesia care unit.

The graphs display the percentages of patients in the intervention group (blue curve) and control group (red curve) achieving total recovery across all postoperative quality of recovery scale (PQRS) domains (physiological, nociceptive, emotional, cognitive and activities of daily living) at each measurement timepoint.

The graphs display the percentages of patients recovered in the intervention (blue curve) and control (red curve) group at each measurement timepoint in the (a) Physiological, (b) nociceptive, (c) emotional (d), cognitive and (e) activities of daily living (ADL) domains of the Postoperative Quality of Recovery Scale (PQRS). Asterisk (∗) indicates a statistically significant difference between groups.

The graphs display the percentages of participants (intervention group blue curve; control group, red curve) reporting: (a) no impact on clarity of thought; (b) mild or no impact on ability to work; (c) mild or no impact on daily living activities and (d) satisfied or totally satisfied with the anaesthetic care, in the overall patient perspective domain of the Postoperative Quality of Recovery Scale (PQRS) at each measurement timepoint. Asterisk (∗) indicates a statistically significant difference between groups.

The additional overall patient perspective domain, with no baseline assessment, revealed that the control group was significantly more satisfied or totally satisfied with the anaesthetic care at 24 h (*P* = 0.017) and 72 (±24)  h (*P* = 0.039) postoperatively (Fig. 5d). Significantly more participants in the control group reported mild impact or no impact in activities of daily living at 14 days (*P* < 0.001; Fig. 5c). In the remaining recovery domains, no differences were observed between the groups over the three-month follow-up period.

### Adverse events

In the control group, one adverse event and one serious adverse event occurred. One participant visited the emergency room for a fever on the first postoperative day but recovered without treatment (Clavien–Dindo grade 1). The serious adverse event involved postoperative bleeding from the gastric anastomosis, requiring reoperation and extended inpatient care; the patient ultimately recovered (Clavien–Dindo grade 3b).

## Discussion

In this multicentre, nonblinded RCT, the opioid-free care pathway combining OFA and HFHI TENS resulted in comparable pain relief to opioid-based standard care but significantly reduced opioid consumption in the PACU and throughout the hospital stay after laparoscopic bariatric surgery. The overall quality of recovery improved in both groups over 3 months. However, significantly more participants in the control group were satisfied with the anaesthetic care in the immediate postoperative period and reported no impact on daily activities 2 weeks after surgery.

Altogether, the study results demonstrate that OFA combined with TENS for postoperative pain management is a viable opioid-sparing concept in bariatric surgery, providing comparable pain relief and recovery outcomes to opioid-based standard treatment for up to 3 months. Three times as many participants achieved adequate pain relief (NRS < 3) in the intervention group despite comparable durations of PACU stay. The similar pain outcomes between groups align with meta-analyses on OFA in various surgical interventions^10,21^ and bariatric surgery specifically,^11^ although the pooled effect indicated significantly lower pain intensity during the PACU stay, favouring OFA,^10,11,21^ none identified a clinically important difference (NRS ≥ 1). Moreover, inconsistencies in the OFA regimens contributed to high heterogeneity, downgrading the level of evidence.

Our intervention incorporating HFHI TENS as the primary pain management strategy following OFA is, to the best of our knowledge, a novel approach not previously described. HFHI TENS after OBA had similar effects on pain intensity and reduced opioid consumption compared to intravenous opioids for postoperative pain in other contexts.^14,15^ Our finding that only 36% of patients in the intervention group required opioids (vs. 90% in the control group), suggests that nearly two-thirds of patients in the intervention group responded to the opioid-free pain management strategy or remained adequately relieved of pain in the PACU. Notably, the preoperative instruction the intervention group received on TENS treatment after randomisation may have positively influenced their postoperative pain experience, as preoperative education about postoperative pain management improves pain outcomes, relieves anxiety and reduces opioid use.^22^ In any case, employing HFHI TENS as a first-line treatment for acute postoperative pain is advantageous, given its cost-effectiveness, absence of serious side effects and the rapid evaluation of its analgesic effect.^15,23^ Intravenous oxycodone has an onset of action of approximately 5 to 8 min,^24^ providing pain relief within 30 min.^25^ Therefore, the 1-min HFHI TENS stimulation regime used in our study could shorten the time-to-pain relief and reduce opioid consumption, or facilitate a swift transition to opioids if the analgesic effect is inadequate.

We observed a significant reduction in opioid consumption in the intervention group during the PACU and in-hospital stay, contrasting with a recent meta-analysis^11^ and systematic review^12^ on OFA in bariatric surgery. Nevertheless, the mean length of PACU stay was similar between the two groups, ensuring that the results were not biased in this regard. The comprehensive design of the intervention, incorporating not only OFA but also HFHI TENS and a dexmedetomidine infusion in the PACU, likely enhanced the opioid-sparing effects. Dexmedetomidine, alone or in multidrug regimens, is the most frequently used opioid substitute for surgical interventions,^10^ including bariatric surgery.^11^ The potential risks of dexmedetomidine at high doses complicate the broader implementation of OFA in clinical practice.^26^ However, the safety profile of OFA using limited doses of dexmedetomidine has been thoroughly evaluated^21^ and is associated with reduced postoperative side effects.^10^ In our OFA regimen, ‘The Gothenburg-model’ (Fig. 1), we used a low dose of dexmedetomidine (0.2 μg kg^−1^ h^−1^) both intraoperatively and postoperatively with no adverse effects in the opioid-free intervention arm, which can be contrasted with a mean dose of 1.2 ± 2 μg kg^−1^ h^−1^ and several serious adverse events reported in the RCT of Beloeil *et al.*^26^

This work builds on the evidence base on OFA in bariatric surgery^11^ and contributes novel long-term quality of recovery data spanning 3 months, addressing a significant knowledge gap highlighted in recent publications.^12,21^ According to the meta-analysis of Feenstra *et al.*,^21^ only 2 of the 38 included studies evaluated quality of recovery, one of which was an RCT in bariatric surgery.^27^ The pooled effect showed that quality of recovery with OFA is superior to OBA up to 24 h postoperatively, and aligns with the results of an RCT on combined opioid-free and loco-regional anaesthesia in bariatric surgery.^28^ In the long-term postbariatric recovery phase, a recently published double-blinded RCT by Clanet *et al.*^29^ showed no significant difference between OFA and OBA in terms of quality of recovery up to 1 month postoperatively, which is consistent with our results. All three RCTs^27–29^ employed a multimodal OFA regimen comparable to our study protocol, apart from one with an additional loco-regional approach.^28^ Yet the outcome on quality of recovery was nonsignificant in our study and that of Clanet *et al.*^29^ Notably, the two RCTs reporting superior early quality of recovery outcomes after OFA did not administer, or at least did not state administering, a bolus dose of a longer acting opioid before emergence from OBA.^27,28^ The omission of a longer acting opioid as preemptive analgesia in the opioid-based groups may have influenced the acute postoperative pain intensity, subsequently affecting early recovery outcomes.

In our study, we did not observe a significant difference in PONV between the groups in the early recovery period or the long-term follow-up. This result differs from previous research showing that OFA reduces the risk of PONV compared to OBA.^10,11,21^ Bariatric surgery itself is a risk factor for PONV,^5^ and there are incentives to avoid inhalational agents to prevent PONV in bariatric surgery, advocating for total intravenous OFA to enhance postoperative recovery.^30^ We saw no difference between groups in cognitive function up to 3 months, which was hypothesised given previous data showing that dexmedetomidine, ketamine and lidocaine may prevent postoperative cognitive dysfunction.^31,32^ Nevertheless, the relatively short duration of anaesthesia for a laparoscopic bariatric procedure, the small sample size and the younger study population (38.8 ± 10.4 years) with few comorbidities do not align with the well documented risk factors for postoperative cognitive dysfunction.^33,34^

The significantly lower satisfaction with anaesthetic care in the intervention group during the first two postoperative days needs to be addressed. According to previous research, the strongest predictor for incomplete satisfaction in the PQRS is the nociceptive domain including persistent pain and PONV on the third postoperative day.^35^ As nociceptive recovery parameters were comparable between groups up to the second postoperative day, this difference in satisfaction could relate to the anaesthetic procedure experience. Our initial publication on this RCT presents a range of patient experiences during OFA induction, with some respondents reporting accentuated memories causing discomfort.^13^ As highlighted by Olausson *et al.*,^13^ healthcare professionals face challenges performing work tasks beyond clinical routine, which may affect the quality of the anaesthetic and postoperative care. The nonroutine use of OFA could have had an impact on bedside presence and thus reduced patient satisfaction with the anaesthetic care. A person-centred care approach that incorporates the entire perioperative care pathway may elevate the quality of care and enhance patient satisfaction by addressing individual needs, fostering better communication between patients and clinicians and ensuring continuity in care from preoperative preparation through postoperative recovery – preferably beyond hospital discharge.^36^ Our finding that a quarter of participants were not fully recovered at 3 months underscores the need for a comprehensive postoperative follow-up approach.^37^

This study has several limitations. The RCT was nonblinded: an open-label design was chosen, as it would be impractical to blind the intervention group receiving HFHI TENS treatment. Knowledge of the allocated treatment may have introduced performance bias, and participants in the intervention group might have avoided postoperative opioids to adhere to the opioid-free protocol, despite the availability of rescue analgesia. A further limitation is that the predefined NRS less than 3 threshold for adequate pain relief no longer reflects current clinical PACU discharge routines, where NRS less than 4 is now accepted. Discharge decisions may also have been influenced by other factors such as patient request, clinical judgement and PACU bed availability, which could explain the relatively high proportion of patients with NRS greater than 3 at discharge in both groups.

Although the intervention encompassed the entire perioperative care pathway, only six participants in the intervention group were excluded from the per-protocol analysis. Moreover, outcome assessments did not differ between groups, the long-term follow-up after 14 days was conducted using an ePRO system, and the analyses were performed by an independent statistician – altogether reducing the influence of detection bias. Furthermore, the extended enrolment period for study participants resulted from disruptions in anaesthesia and surgical operations at both hospitals owing to the COVID-19 pandemic. This caused a notable reduction in the number of bariatric procedures performed between 2021 and 2022.

Another limitation is loss to follow-up, as 14.5% of participants (eight per group), did not complete the 3-month follow-up, which could introduce attrition bias concerning the secondary long-term outcomes. Nevertheless, the randomisation procedure was thorough, resulting in comparable baseline characteristics. Contrasting with other RCTs evaluating OFA, our control group received a multimodal, yet opioid-based treatment, which does not exaggerate the effects of the opioid-free intervention. Our long-term follow-up should be considered a strength, as previous studies have highlighted such outcomes as a knowledge gap.^12,21^

The effectiveness of OFA combined with HFHI TENS demonstrates the feasibility of this concept in bariatric surgery, underscoring its clinical importance given the need for opioid-sparing strategies.^9^ This innovative approach has the potential to eliminate perioperative opioid use, providing an alternative for patients at high risk of opioid-related adverse effects and accommodating individual needs and pain management preferences. OFA should be recognised as a novel approach requiring education and training for clinicians and patients to ensure successful clinical implementation.^38,39^ Further refinements of the care pathway may be considered, such as person-centred care interventions and pharmacological protocol adjustments, given the observed differences in satisfaction and experiences of OFA induction.^13^

## Conclusion

This multicentre, nonblinded RCT demonstrates that a novel opioid-free care pathway provides comparable pain relief and long-term recovery outcomes to opioid-based standard care after laparoscopic bariatric surgery, while significantly reducing opioid consumption throughout the PACU and in-hospital stay. The authors encourage clinicians and policymakers to consider a novel opioid-free approach encompassing the entire perioperative period.

## Supplementary Material

Supplemental Digital Content

## References

[1] Kolotkin RL Davidson LE Crosby RD. Six-year changes in health-related quality of life in gastric bypass patients versus obese comparison groups. *Surg Obes Relat Dis* 2012; 8:625–633. 22386053 10.1016/j.soard.2012.01.011 PMC3693474

[2] Sjöström L Lindroos AK Peltonen M. Lifestyle, diabetes, and cardiovascular risk factors 10 years after bariatric surgery. *N Engl J Med* 2004; 351:2683–2693. 15616203 10.1056/NEJMoa035622

[3] D’Hondt M Vanneste S Pottel H. Laparoscopic sleeve gastrectomy as a single-stage procedure for the treatment of morbid obesity and the resulting quality of life, resolution of comorbidities, food tolerance, and 6-year weight loss. *Surg Endosc* 2011; 25:2498–2504. 21359900 10.1007/s00464-011-1572-x

[4] Kositanurit W Muntham D Udomsawaengsup S. Prevalence and associated factors of obstructive sleep apnea in morbidly obese patients undergoing bariatric surgery. *Sleep Breath* 2018; 22:251–256. 28396972 10.1007/s11325-017-1500-y

[5] Kushner BS Freeman D Sparkman J. Assessment of postoperative nausea and vomiting after bariatric surgery using a validated questionnaire. *Surg Obes Relat Dis* 2020; 16:1505–1513. 32665115 10.1016/j.soard.2020.05.017

[6] Hartwig M Allvin R Bäckström R. Factors associated with increased experience of postoperative pain after laparoscopic gastric bypass surgery. *Obes Surg* 2017; 27:1854–1858. 28144798 10.1007/s11695-017-2570-4 PMC5489569

[7] Svensson CJ Lundberg CE Sandström TZ. Opioid consumption in patients undergoing Roux-en-Y bariatric surgery compared with population controls with and without obesity. *Surg Obes Relat Dis* 2022; 18:107–116. 34493454 10.1016/j.soard.2021.08.010

[8] Raebel MA Newcomer SR Reifler LM. Chronic use of opioid medications before and after bariatric surgery. *JAMA* 2013; 310:1369–1376. 24084922 10.1001/jama.2013.278344

[9] Stenberg E dos Reis Falcão LF O’Kane M. Guidelines for perioperative care in bariatric surgery: enhanced recovery after surgery (ERAS) Society recommendations: a 2021 update. *World J Surg* 2022; 46:729–751. 34984504 10.1007/s00268-021-06394-9 PMC8885505

[10] Olausson A Svensson CJ Andréll P. Total opioid-free general anaesthesia can improve postoperative outcomes after surgery, without evidence of adverse effects on patient safety and pain management: A systematic review and meta-analysis. *Acta Anaesthesiol Scand* 2021; 66:170–185. 34724195 10.1111/aas.13994

[11] Hung KC Chiu CC Hsu CW. Impact of opioid-free anesthesia on analgesia and recovery following bariatric surgery: a meta-analysis of randomized controlled studies. *Obes Surg* 2022; 32:3113–3124. 35854095 10.1007/s11695-022-06213-7

[12] Mieszczański P Kołacz M Trzebicki J. Opioid-free anesthesia in bariatric surgery: is it the one and only? A comprehensive review of the current literature. *Healthcare (Basel)* 2024; 12:1094. 38891169 10.3390/healthcare12111094 PMC11171472

[13] Olausson A Angelini E Heckemann B. Patients’ perioperative experiences of an opioid-free versus opioid-based care pathway for laparoscopic bariatric surgery: a qualitative study. *Int J Nurs Stud Adv* 2024; 6:100201. 38746814 10.1016/j.ijnsa.2024.100201 PMC11080373

[14] Piasecki A Ögren C Thörn SE. High-frequency, high-intensity transcutaneous electrical nerve stimulation compared with opioids for pain relief after gynecological surgery: a systematic review and meta-analysis. *Scand J Pain* 2023; 24: 10.1515/sjpain-2023-0068 37819201

[15] Ögren C Varkey E Wolf A. High-frequency, high-intensity TENS compared to standard treatment with opioids for postoperative pain relief after laparoscopic cholecystectomy: a multicentre randomized controlled trial. *Eur J Pain* 2024; 28:1772–1784. 38943342 10.1002/ejp.2308

[16] Berdine HJ Nesbit SA. Equianalgesic dosing of opioids. *J Pain Palliat Care Pharmacother* 2006; 20:79–84. 17182514

[17] Royse CF Newman S Chung F. Development and feasibility of a scale to assess postoperative recovery: the postoperative quality recovery scale. *Anesthesiology* 2010; 113:892–905. 20601860 10.1097/ALN.0b013e3181d960a9

[18] Jildenstål P Eriksson J Warren Stomberg M. Evaluation of the Postoperative Quality of Recovery Scale test and re-test in Swedish among healthy volunteers. *F1000Res* 2016; 5:2549. 28299175 10.12688/f1000research.9740.1 PMC5310376

[19] Clavien PA Barkun J de Oliveira ML. The Clavien-Dindo classification of surgical complications: five-year experience. *Ann Surg* 2009; 250:187–196. 19638912 10.1097/SLA.0b013e3181b13ca2

[20] Platon B Andréll P Raner C. High-frequency, high-intensity transcutaneous electrical nerve stimulation as treatment of pain after surgical abortion. *Pain* 2010; 148:114–119. 19959293 10.1016/j.pain.2009.10.023

[21] Feenstra ML Jansen S Eshuis WJ. Opioid-free anesthesia: a systematic review and meta-analysis. *J Clin Anesth* 2023; 90:111215. 37515877 10.1016/j.jclinane.2023.111215

[22] Darville-Beneby R Lomanowska AM Yu HC. The impact of preoperative patient education on postoperative pain, opioid use, and psychological outcomes: a narrative review. *Can J Pain* 2023; 7:2266751. 38126044 10.1080/24740527.2023.2266751 PMC10732618

[23] Johnson MI Paley CA Jones G. Efficacy and safety of transcutaneous electrical nerve stimulation (TENS) for acute and chronic pain in adults: a systematic review and meta-analysis of 381 studies (the meta-TENS study). *BMJ Open* 2022; 12:e051073. 10.1136/bmjopen-2021-051073 PMC8845179 35144946

[24] Pergolizzi JV Jr Seow-Choen F Wexner SD. Perspectives on intravenous oxycodone for control of postoperative pain. *Pain Pract* 2016; 16:924–934. 26393529 10.1111/papr.12345

[25] Raff M Belbachir A El-Tallawy S. Intravenous oxycodone versus other intravenous strong opioids for acute postoperative pain control: a systematic review of randomized controlled trials. *Pain Ther* 2019; 8:19–39. 31004317 10.1007/s40122-019-0122-4 PMC6514019

[26] Beloeil H Garot M Lebuffe G. POFA Study Group, SFAR Research Network. Balanced opioid-free anesthesia with dexmedetomidine versus balanced anesthesia with remifentanil for major or intermediate noncardiac surgery. *Anesthesiology* 2021; 134:541–551. 33630043 10.1097/ALN.0000000000003725

[27] Mulier JP Wouters R Dillemans B. A randomized controlled, double-blind trial evaluating the effect of opioid-free versus opioid general anaesthesia on postoperative pain and discomfort measured by the QoR-40. *J Clin Anesth Pain Med* 2018; 6:2.

[28] Ibrahim M Elnabtity AM Hegab A. Combined opioid free and loco-regional anaesthesia enhances the quality of recovery in sleeve gastrectomy done under ERAS protocol: a randomized controlled trial. *BMC Anesthesiol* 2022; 22:29. 35062872 10.1186/s12871-021-01561-w PMC8781357

[29] Clanet M Touihri K El Haddad C. Effect of opioid-free versus opioid-based strategies during multimodal anaesthesia on postoperative morphine consumption after bariatric surgery: a randomised double-blind clinical trial. *BJA Open* 2024; 9:100263. 38435809 10.1016/j.bjao.2024.100263 PMC10906147

[30] Ziemann-Gimmel P Schumann R English W. Preventing nausea and vomiting after bariatric surgery: is the Apfel risk prediction score enough to guide prophylaxis? *Obes Surg* 2020; 30:4138–4140. 32415631 10.1007/s11695-020-04682-2

[31] Li M Yang Y Ma Y. Pharmacological agents that prevent postoperative cognitive dysfunction in patients with general anesthesia: a network meta-analysis. *Am J Ther* 2020; 28:e420–e433. 34228651 10.1097/MJT.0000000000001271

[32] Geng C Hu B Jiang J. The effect of intravenous lidocaine on postoperative cognitive dysfunction: a systematic review and meta-analysis. *BMC Anesthesiol* 2023; 23:299. 37670239 10.1186/s12871-023-02202-0 PMC10478315

[33] Moller JT Cluitmans P Rasmussen LS. Long-term postoperative cognitive dysfunction in the elderly ISPOCD1 study. ISPOCD investigators. International Study of Post-Operative Cognitive Dysfunction. *Lancet* 1998; 351:857–861. 9525362 10.1016/s0140-6736(97)07382-0

[34] Monk Terri G Weldon BC Garvan Cyndi W. Predictors of cognitive dysfunction after major noncardiac surgery. *Anesthesiology* 2008; 108:18–30. 18156878 10.1097/01.anes.0000296071.19434.1e

[35] Royse CF Chung F Newman S. Predictors of patient satisfaction with anaesthesia and surgery care: a cohort study using the Postoperative Quality of Recovery Scale. *Eur J Anaesthesiol* 2013; 30:106–110. 22907610 10.1097/EJA.0b013e328357e584

[36] Arakelian E Swenne CL Lindberg S. The meaning of person-centred care in the perioperative nursing context from the patient's perspective - an integrative review. *J Clin Nurs* 2017; 26:2527–2544. 27862496 10.1111/jocn.13639

[37] Schwoerer A Kasten K Celio A. The effect of close postoperative follow-up on co-morbidity improvement after bariatric surgery. *Surg Obes Relat Dis* 2017; 13:1347–1352. 28501321 10.1016/j.soard.2017.03.024

[38] Forget P Van de Velde M Pogatzki-Zahn E. Opioid-free anaesthesia: should we all adopt it? An overview of current evidence. *Eur J Anaesthesiol* 2023; 40:539–541. 37405716 10.1097/EJA.0000000000001775

[39] Blum KA Liew LY Dutia AR. Opioid-free anesthesia: a practical guide for teaching and implementation. *Minerva Anestesiol* 2024; 90:300–310. 38482635 10.23736/S0375-9393.23.17824-2

