# Supplementary material for: Effects of an opioid-free care pathway vs. opioid-based standard care on postoperative pain and postoperative quality of recovery after laparoscopic bariatric surgery: A multicentre randomised controlled trial
Source: Eur J Anaesthesiol. 2025 May 14;42(8):714–26. doi: 10.1097/EJA.0000000000002193 (PMC12237137; doi:10.1097/EJA.0000000000002193)
Supplement: Supplemental Digital Content [file ejanet-42-714-s001.docx]

**Supplement 1** Calculation of the PQRS score for the statistical analysis

The Postoperative Quality of Recovery Scale (PQRS) score was calculated by subtracting the postoperative value from the baseline value in each parameter, and the performance was dichotomized as either 'recovered' or 'not recovered' at each postoperative time point within each domain. A subject was considered 'recovered' if the postoperative value was equal to or greater than the baseline value. Total recovery was defined as the participant’s returning to baseline values or better across all domains and all parameters in the PQRS tool.^1^ An exception was made for the cognitive dimension. The permissible changes from baseline scores are as follows: ≥ 0 for 'orientation', ≥ -2 for 'digits forward', ≥ -1 for 'digits backward', ≥ -3 for 'word recall', and ≥ -3 for 'word generation'. This allowance accounts for natural variations in cognitive performance; thus, a participant may exhibit slight declines in attention and concentration compared to baseline but still be classified as recovered.^2^

**References**

1. Royse CF, Newman S, Chung F et al. Development and feasibility of a scale to assess postoperative recovery: the post-operative quality recovery scale. *Anesthesiology* 2010; **113**:892-905.

2. Royse CF, Newman S, Williams Z, Wilkinson DJ. A human volunteer study to identify variability in performance in the cognitive domain of the postoperative quality of recovery scale. *Anesthesiology* 2013; **119**:576-581.
